# Intradialytic Complement Activation Precedes the Development of Cardiovascular Events in Hemodialysis Patients

**DOI:** 10.3389/fimmu.2018.02070

**Published:** 2018-09-13

**Authors:** Felix Poppelaars, Mariana Gaya da Costa, Bernardo Faria, Stefan P. Berger, Solmaz Assa, Mohamed R. Daha, José Osmar Medina Pestana, Willem J. van Son, Casper F. M. Franssen, Marc A. Seelen

**Affiliations:** ^1^Division of Nephrology, Department of Internal Medicine, University of Groningen, University Medical Center Groningen, Groningen, Netherlands; ^2^Nephrology and Infecciology Group, INEB/I3S, University of Porto, Porto, Portugal; ^3^Department of Nephrology, Hospital Braga, Braga, Portugal; ^4^Department of Cardiology, University of Groningen, University Medical Center Groningen, Groningen, Netherlands; ^5^Department of Nephrology, University of Leiden, Leiden University Medical Center, Leiden, Netherlands; ^6^Nephrology Division, Federal University of São Paulo, São Paulo, Brazil

**Keywords:** complement, kidney, cardiovascular risk, hemodialysis, biocompatibility, innate immunity, C1-inhibitor

## Abstract

**Background:** Hemodialysis (HD) is a life-saving treatment for patients with end stage renal disease. However, HD patients have markedly increased rates of cardiovascular morbidity and mortality. Previously, a link between the complement system and cardiovascular events (CV-events) has been reported. In HD, systemic complement activation occurs due to blood-to-membrane interaction. We hypothesize that HD-induced complement activation together with inflammation and thrombosis are involved in the development of CV-events in these patients.

**Methods:** HD patients were followed for the occurrence of CV-events during a maximum follow-up of 45 months. Plasma samples were collected from 55 patients at different time points during one HD session prior to follow-up. Plasma levels of mannose-binding lectin, properdin and C3d/C3 ratios were assessed by ELISA. In addition, levels of von Willebrand factor, TNF-α and IL-6/IL-10 ratios were determined. An *ex-vivo* model of HD was used to assess the effect of complement inhibition.

**Results:** During median follow-up of 32 months, 17 participants developed CV-events. In the CV-event group, the C3d/C3-ratio sharply increased 30 min after the start of the HD session, while in the event-free group the ratio did not increase. In accordance, HD patients that developed a CV-event also had a sustained higher IL-6/IL-10-ratio during the first 60 min of the HD session, followed by a greater rise in TNF-α levels and von Willebrand factor at the end of the session. In the *ex-vivo* HD model, we found that complement activation contributed to the induction of TNF-α levels, IL-6/IL-10-ratio and levels of von Willebrand factor.

**Conclusions:** In conclusion, these findings suggest that early intradialytic complement activation predominantly occurred in HD patients who develop a CV-event during follow-up. In addition, in these patients complement activation was accompanied by a pro-inflammatory and pro-thrombotic response. Experimental complement inhibition revealed that this reaction is secondary to complement activation. Therefore, our data suggests that HD-induced complement, inflammation and coagulation are involved in the increased CV risk of HD patients.

## Introduction

Renal replacement therapy (RRT) represents a cornerstone in the treatment of patients with end stage renal disease (ESRD). Hemodialysis (HD) remains the most common form of RRT (1). Despite being lifesaving, HD comes with a risk (2). The life expectancy and quality of life of patients on dialysis is inferior to the general population. Overall, HD has been associated with increased cardiovascular morbidity and mortality (3). Previous studies have suggested that the innate immune system plays a key role in the development of cardiovascular disease in HD patients (4).

The complement system is a major component of innate immunity and activation of this system induces an inflammatory response (5). Complement activation can occur via three pathways: the classical pathway (CP), lectin pathway (LP), and alternative pathway (AP). Regardless of the trigger, all pathways lead to the cleavage of C3 resulting in the formation of C3b, the large fragment and C3a, an anaphylatoxin. Ultimately, C3b is broken down progressively to iC3b and then to the more stable fragment C3d. The functions of the complement system were thought to be limited to opsonization and lysis of pathogens. However, nowadays this system is known to have numerous functions and complement has been shown to be involved in the pathogenesis of various diseases (6).

For decades, HD has been known to activate the complement system (7). In dialysis, complement activation is mainly caused by the interaction of blood with the HD membrane (4). Regardless of the efforts to improve biocompatibility, complement activation still occurs in HD, even with modern membranes (8–10). It has been hypothesized that complement activation leads to HD-induced inflammation and thereby increases the subsequent cardiovascular risk (4). In accordance, several studies have shown an association between complement and cardiovascular events (CV-event) (8, 11–14). However, the link between complement activation products and CV-events remains poorly characterized (15). Only Lines et al. reported an association in HD patients between soluble C5b-9 and cardiovascular risk (15). Furthermore, previous experimental studies proposed a link between HD-induced complement activation, pro-inflammatory cytokines, and the coagulation system (10, 16).

We hypothesize that an unfavorable complement profile is seen in HD patients who will develop a CV-event. To investigate the mechanism of increased cardiovascular risk in HD, we measured complement activation, pro-inflammatory cytokines and pro-thrombotic factors during one HD session in patients that developed a CV-event during follow-up and compared this to patients without a CV-event during follow-up. Furthermore, we used an *ex-vivo* model of HD to further elucidate the role of complement activation as a trigger for inflammation and coagulation in HD.

## Materials and methods

### Study population and design

A cohort of 55 hemodialysis patients from Dialysis Center Groningen and the University Medical Center Groningen were followed for a maximum of 45 months. The original cohort was composed out of 109 patients; however, due to a lack of samples only 55 patients could be included for this study. The protocol has been previously described (2). In short, patients were included if the duration of HD therapy was longer than 3 months. Patients with severe heart failure (NYHA class IV) were excluded. Patient characteristics were extracted from patient records.

### Dialysis settings

Patients were on maintenance HD treatment for three times a week with a low-flux polysulfone hollow-fiber dialyzer (F8; Fresenius Care, Bad Homburg, Germany). The hemodialysis sessions lasted for 4 h. The blood and dialysate flow rates were 250–350 and 500 mL/min, respectively. A constant ultrafiltration rate was used. Dialysate composition was as follows: acetate, 3.0 mmol/L; bicarbonate, 34 mmol/L; calcium, 1.5 mmol/L; chloride, 108 mmol/L; glucose, 1.0 g/L; magnesium, 0.5 mmol/L; potassium, 1.0 or 2.0 mmol/L; sodium, 139 mmol/L The dialysate temperature was kept on 36.0 or 36.5°C. Blood samples were taken just before the start of the dialysis session, and after 30, 60, 180, and 240 min.

### Definition of endpoint

The end-point of the study was defined as the time to the first CV-event. CV-events included cardiac, cerebrovascular, or peripheral vascular events. Occurrence of a cardiac event was defined as a ischemic heart disease (unstable angina pectoris, myocardial infarction, Coronary Artery Bypass Grafting (CABG) and/or Percutaneous Coronary Intervention (PCI), sudden cardiac death and congestive heart failure. In order to classify as acute myocardial infarction, two out of the following three criteria had to be present: clinical status, elevated heart enzymes, and EKG changes. Cerebrovascular events were defined as stroke, ischemic insult, or newly diagnosed >70% stenosis of the extracranial carotid artery. Strokes and ischemic insults had to be verified by CT or MRI. Peripheral vascular disease was defined as having intermittent claudication with angiographically or sonographically proven stenosis >50% of the major arteries of the lower limbs or ulcers caused by atherosclerotic stenosis or surgery for this disorder. Transplantation was a censoring event and the transplantation date was considered as the final follow-up date (17).

### *Ex-vivo* model of hemodialysis

An *ex-vivo* model of HD was used as previously described (18). In brief, a closed circuit was assembled using a pediatric polysulfone hollow-fiber dialyzer (FX paed; Fresenius Care, Germany) and blood lines (SN-Set ONLINEplus BVM 5008-R, Fresenius Care, Germany). The total volume of the circuit was approximately 50 mL. Perfusion was achieved using a Masterflex® peristaltic pump (Cole-Parmer, USA) and was flow-controlled (TS410 tubing flow module, Transonic systems Inc, USA) to reach a perfusion flow of approximately 140 to 160 mL/min. The temperature was kept constant at 37°C and controlled by an external heater. Whole blood was taken from healthy volunteers (*n* = 3) and anticoagulated with low-molecular weight heparin (1 U/mL). Initially, the circuit was primed with NaCl 0.9% and perfused for approximately 20 min to remove air bubbles. Prior to perfusion, the dialysate compartment was filled with NaCl 0.9% and closed. Next, freshly drawn heparinized blood was added to the circuit, while the same volume of the saline solution was discarded. The system was perfused with recirculating blood for 4 h. Samples were collected at the start of the perfusion and after 30, 60, 120, 180, and 240 min. To investigate the effect of complement inhibition, two consecutive sessions were performed for each healthy volunteer. During one session, 200 units of C1-inhibitor (Cinryze, Viropharma, USA) were added to the blood prior to perfusion, whereas the other session without C1-inhibitor (C1-INH) served as a control.

### Inflammatory markers and pro-thrombotic factors

In the HD cohort, TNF-α was measured by Quantikine HS Human Immunoassay (R&D System Inc., USA). Furthemore, IL-6 and IL-10 were determined using a quantitative sandwich enzyme immunoassay technique (R&D System Inc., USA). Lastly, Von Willebrand Factor (vWF) was measured by enzyme-linked immunosorbent assay (Dakopatts, UK). In the *ex-vivo* HD model, TNF-α, IL-6, IL-10, and vWF were measured using a human magnetic luminex assay (R&D Systems Inc, USA) according to the manufacturer's instructions.

### Quantification of complement proteins

C3d was measured by sandwich enzyme immunoassay as previously described (19). Quantitative antigenic assay for C3 was performed by the radial immunodiffusion technique with monospecific anti-sera (19). The C3d/C3 ratio was determined by dividing the C3d values in μg/mL by the C3 concentration in mg/mL. Additionally, Properdin and MBL concentrations were measured as described earlier (19, 20).

### Statistics

Statistical analysis was performed using IBM SPSS 22.0 (IBM Corporation, Chicago, IL, USA). Normally distributed data are presented as mean ± standard deviation, whereas non-normally distributed data are shown as median with interquartile range. Nominal data are displayed as total number of patients with percentage [*n* (%)]. Differences between two groups were assessed with the student *t*-test, whereas the paired *t*-test was used to compare values of a single variable during different time points within the HD session. A one-way ANOVA was used when assessing for differences in multiple groups, followed by Bonferroni's *post-hoc* comparisons tests. The association between different variables and the incidence of CV-event were assessed by Cox proportional hazard regression. The Harrell's C statistic is the equivalent of the area under the ROC curve, if the outcome is binary (21).

### Ethics

This study was performed in accordance to the Declaration of Helsinki and was approved by the Medical Ethical Committee from the University Medical Center Groningen. All participants signed informed written consent.

## Results

### Patients characteristics

Blood samples from 55 patients on maintenance HD were available, of which 35 were male and 20 female (Table 1). The mean age was 62 ± 15 years and baseline dialysis vintage was 1.2 years [IQR: 0.6–3.9 years]. The median follow-up of the study was 32 months and during this time 17 patients (31%) developed a CV-event, whereas 16 patients died (29%). In our study, the causes of death were cardiovascular (44%), infection (12.5%), discontinuation of the HD treatment (12.5%), or unknown (31%). Among the patients that developed CV-events, 35% had acute coronary syndrome, 17% needed coronary artery bypass surgery, 11% developed congestive heart failure, 17% had a cerebro-vascular accident and 17% developed peripheral vascular disease. Next, we created two different groups; the 17 patients that developed a CV-event during follow-up (CV-event group) and the 38 patients that remained event-free (event-free group).

**Table 1:** Baseline characteristics of our study population of hemodialysis patients with and without a cardiovascular event.

		**Patients**			**Univariable**	
	**All (*n* = 55)**	**CV-event (*n* = 17)**	**No CV-event (*n* = 38)**	P*	**St. beta**	***P*^#^**
**C3d/C3 RATIO**						
0 min	5.9 [4.8–9.3]	5.6 [4.7–8.1]	6.1 [4.8–11.5]	0.2	−0.02	0.6
30 min	9.8 [7.3–17.0]	12.0 [10–33.5]	8.4 [6.8–13.4]	**0.01**	0.05	**0.002**
60 min	8.4 [5.4–12.4]	7.8 [4.4–11.9]	8.9 [5.6–16.2]	0.2	−0.04	0.2
120 min	8.6 [5.5–14.5]	7.4 [5.2–12.7]	9.1 [5.6–16.4]	0.3	−0.03	0.3
240 min	8.4 [5.8–17.9]	8.6 [5.9–20.0]	8.3 [5.8–17.9]	0.8	−0.04	0.8
**DEMOGRAPHICS**						
Age, years	62.7 ± 15.8	66.5 ± 11.4	61.0 ± 17.2	0.2	0.01	0.3
Male gender, n (%)	35 (63)	11 (64)	24 (63)	0.9	−0.22	0.6
Current diabetes, n (%)	10 (18)	5 (29)	5 (13)	0.2	0.51	0.3
Hypertension, n (%)	48 (87)	14 (82)	34 (89)	0.2	−0.99	0.1
Cardiovascular history, n (%)	11 (20)	5 (29)	6 (15)	0.2	0.64	0.2
BSA, m^2^	1.8 ± 0.3	1.7 ± 0.5	1.9 ± 0.2	0.08	−2.04	**0.02**
**HEMODIALYSIS**						
Dialysis vintage, months	14.1 [7.3–47.3]	12.2 [8.6–50.4]	16.4 [4.7–47.5]	0.5	0.002	0.8
Ultrafiltration volume, L	2.5 ± 0.7	2.4 ± 0.6	2.5 ± 0.7	0.9	0.00	0.7
Ultrafiltration rate, ml/kg/h	8.1 ± 2.3	7.9 ± 1.8	8.2 ± 2.5	0 7	−0.01	0.8
**PRIMARY RENAL DISEASE**, ***n*** **(%)**						
Hypertension	12 (22)	4 (23)	8 (21)	0.8	0.02	0.9
Diabetes	5 (9)	3 (17)	2 (5)	0.1	0.65	0.3
ADPKD	7 (13)	1 (6)	6 (16)	0.3	−0.96	0.3
FSGS	6 (11)	2 (12)	4 (10)	0.8	0.89	0.2
IgA nephropathy	3 (6)	0 (0)	3 (7)	0.2	−3.15	0.4
Chronic pyelonephritis	1 (2)	1 (6)	0 (0)	0.1	2.02	0.06
Glomerulonephrytis	6 (11)	1 (6)	5 (13)	0.4	−0.75	0.4
Other Diagnosis	9 (16)	3 (18)	6 (16)	0.8	0.35	0.6
Unknown	5 (9)	2 (12)	3 (7)	0.8	−0.34	0.9
**LABORATORY MEASUREMENTS**						
Hematocrit, %	34.4 ± 3.7	34.0 ± 4.2	34.6 ± 3.5	0.6	−5.98	0.3
HbA1C, mmol/mol	5.56 ± 0.9	5.7 ± 1.1	5.5 ± 0.8	0.4	0.17	0.4
Albumin, g/L	38 [37-41]	38 [37-41]	38 [36-42]	0.9	0.004	0.9
Calcium, mmol/L	2.32 ± 0.2	2.31 ± 0.1	2.32 ± 0.2	0.8	−0.55	0.6
Phosphate, mmol/L	1.74 ± 0.5	1.70 ± 0.4	1.75 ± 0.6	0.7	0.009	0.9
hsCRP, mg/L	5.5 [1.5–8.8]	5.0 [2.3–11.8]	5.8 [1.4–8.8]	0.5	0.01	0.3
**MEDICATION**						
Aspirin, n (%)	34 (62)	8 (47)	26 (68)	0.2	−0.80	0.09
Calcium channel blockers, n (%)	12 (22)	4 (23)	8 (21)	0.8	−0.14	0.7
β-Blocker, n (%)	31 (56)	10 (59)	21 (55)	0.8	0.24	0.6
ACE inhibitor, n (%)	5 (9)	1 (5)	4 (10)	0.6	−0.44	0.6
AT2–receptor antagonists, n (%)	8 (14)	2 (12)	6 (16)	0.7	−0.41	0.6
Statin, n (%)	13 (24)	2 (12)	11 (29)	0.2	−0.88	0.2
Diuretics, n (%)	5 (9)	2 (12)	3 (7)	0.7	1.11	0.2

P* indicates P-value for the difference in baseline characteristics between the patient with and without a cardiovascular-event. Differences were tested by Student's t-Test or Mann–Whitney U test for continuous variables and with χ^2^ test for categorical variables. Data are presented as mean ± SD or median [IQR]. P^#^ indicates P-value for univariate Cox-regression for the occurrence of CV-event. Data are presented as beta coefficient with corresponding P-value.

*CV, cardiovascular; BSA, body surface area; ADPKPD, autosomal dominant polycystic disease; FSGS, focal segmental glomerulosclerosis; HbA1c, Hemoglobin A1c; hsCRP, high sensitive C-reactive protein; ACE inhibitor, angiotensin-converting-enzyme inhibitor; AT2-receptor antagonists, Angiotensin II receptor antagonists*.

### Complement activation in the HD patients

**Figure 1:**
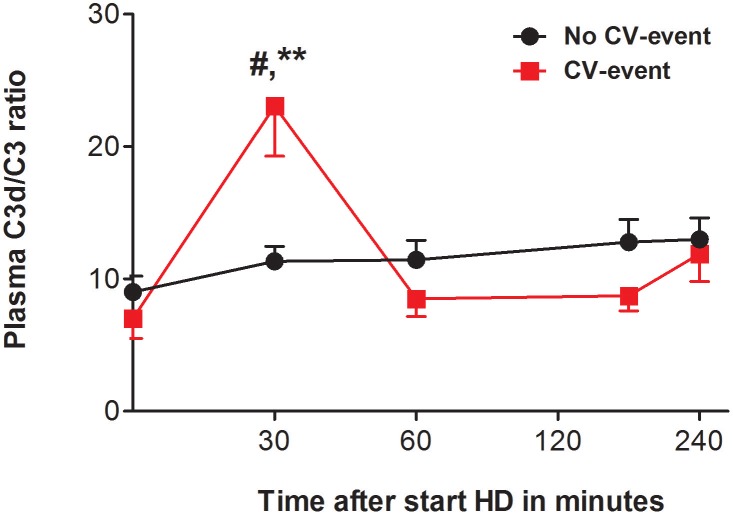
The C3d/C3-ratio during hemodialysis. Course of plasma C3d/C3 ratio in patients that developed a cardiovascular event (CV-event) during follow-up and in those that remained CV-event free (no CV-event). The data is presented as mean ± SEM and C3d/C3-ratio was calculated by dividing the C3d values (μg/mL) by the C3 levels (in mg/mL). The C3d/C3-ratio was determined at the start of hemodialysis session and 30, 60, 180 and 240 min after. Differences between the two groups were assessed by the student *t*-test and a one-way ANOVA followed by Bonferroni's *post-hoc* comparisons tests was used to compare C3d/C3 ratios at different time points within one group (**P < 0.01). The hashtag above the bars denotes a significant difference between the two groups (^#^*P* < 0.05), whereas the asterisk above the bars denotes a significant difference compared to baseline within the group. The number of subject is 17 in the “CV-event group” and 38 in the “No CV-event group”.

To assess complement activation, we determined the C3d/C3-ratio in 55 patients during one HD session at the start of the follow-up. The C3d/C3-ratio at baseline was not statistically different between the patients that would develop a CV-event (7.0 ± 6.2) compared to the patients that would not (9.0 ± 7.4). Surprisingly, at the end of the HD session the C3d/C3-ratio was also not statistically different between the two groups (CV-event group: 11.8 ± 8.5, event-free group: 12.9 ± 10.0). However, when the intradialytic C3d/C3-ratios were compared between the two groups, clear differences were seen (Figure 1 and Table 1). At 30 min intradialysis, there was a significant increase in the C3d/C3-ratio in the CV-event group compared to the patients who remained event-free. During these initial 30 min, the C3d/C3-ratio increased by 3.29 fold in the CV-event group and by only 1.26 fold in the event-free group (*P* < 0.01). In addition, Cox regression analysis was performed to assess the association between C3d/C3 ratio at 30 min and occurrence of a CV-event (Table 2). In the crude model, C3d/C3 ratios were associated with a hazard ratio of 1.06 (95% CI 1.02-1.09; *P* < 0.001). After adjustment for age and gender, variables with *P* < 0.1 in univariate analysis (BSA, chronic pyelonephritis as primary renal disease and use of aspirin), cardiovascular risk factors (CV history, DM and hypertension) or characteristics of HD (Ultrafiltration rate, ultrafiltration volume and dialysis vintage), the association between C3d/C3 ratio at 30 min and CV-event remained significant. Subsequently, the Harrell's-C statistics was determined to further confirm the potential relationship between complement activation and CV-events. Plasma C3d/C3 ratio at 30 min had a Harrell's-C statistics of 0.71 (95% CI 0.55–0.88; *P* = 0.01).

**Table 2:** Associations of intradialytic complement activation with outcome.

	**Cardiovascular events**		
	**C3d/C3 ratio at 30 min**		
	**HR**	**95% CI**	***P***
Model 1	1.06	1.02–1.09	< 0.001
Model 2	1.06	1.03–1.09	0.001
Model 3	1.04	1.01–1.08	0.03
Model 4	1.06	1.02–1.09	0.001
Model 5	1.07	1.02–1.09	0.002

*Model 1: crude. Model 2: adjusted for age and gender. Model 3: adjusted for BSA, aspirin and primary chronic pyelonephritis. Model 4: adjusted for DM, cardiovascular history and hypertension. Model 5: adjusted for HD vintage, UF rate and UF volume*.

*Data are presented as hazard ratio (HR) plus 95% confidence interval (CI). BSA, body surface area; DM, diabetes mellitus; HD, Hemodialysis; UF, ultrafiltration rate*.

**Figure 2:**
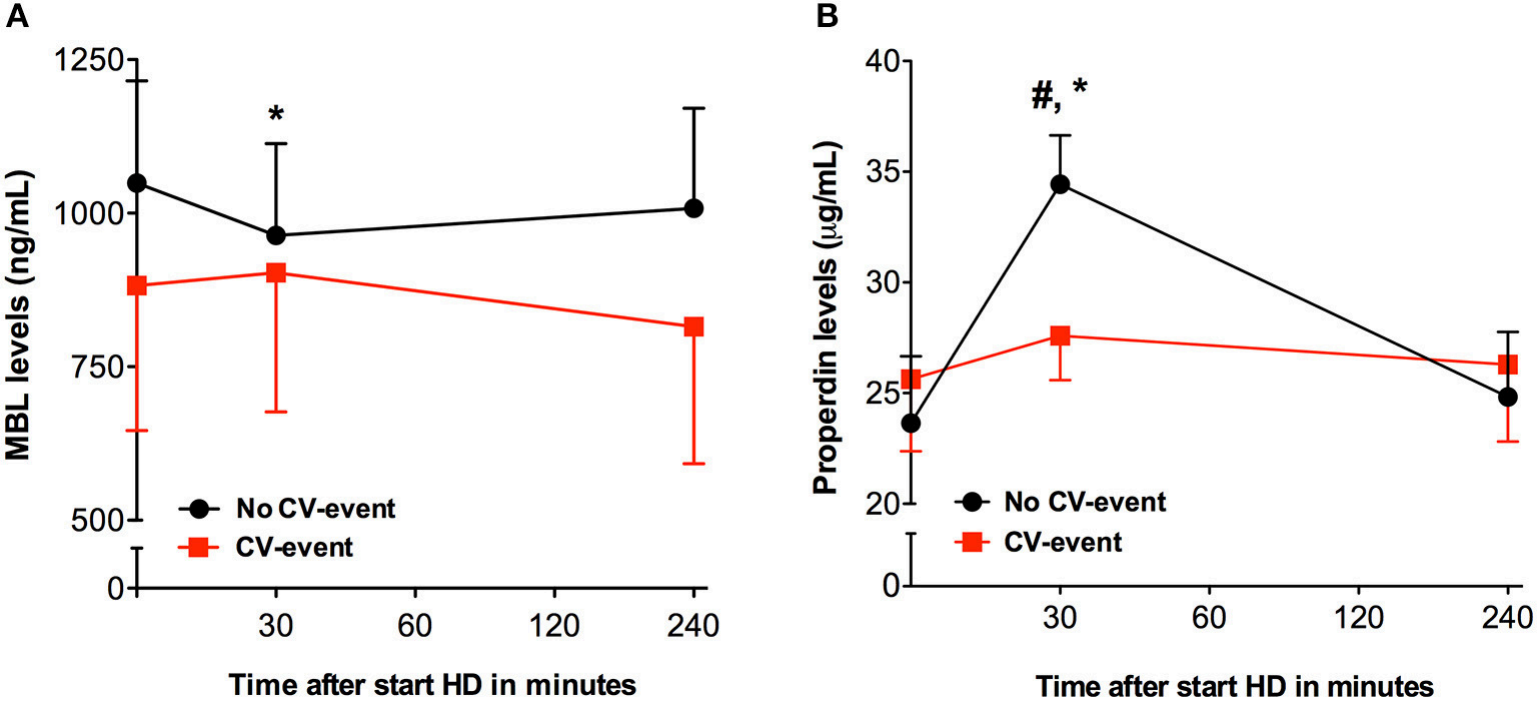
Intradialytic levels of properdin and Mannose-binding lectin. Course of plasma mannose-binding lectin (MBL) and properdin in patients that developed a cardiovascular event (CV-event) during follow-up and in those that remained CV-event free (no CV-event). The data is presented as mean ± SEM. **(A)** The levels of MBL were determined at the start of hemodialysis session and 30 and 240 min after. **(B)** The levels of properdin were determined at the start of hemodialysis session and 30 and 240 min after. Differences between the two groups were assessed by the student *t*-test and a one-way ANOVA followed by Bonferroni's *post-hoc* comparisons tests was used to compare levels at different time points within one group (*P < 0.05). The hashtag above the bars denotes a significant difference between the two groups (^#^*P* < 0.05), whereas the asterisk above the bars denotes a significant difference compared to baseline within the group. The number of subject is 11 in the “CV-event group” and 19 in the “No CV-event group”.

We next set out to assess the contribution of the AP and LP to HD-induced complement activation. Due to a lack of samples, properdin and MBL levels were measured in a subgroup of 30 patients (Figure 2). In this subgroup, there were 11 patients in the CV-event group and 19 patients in the event free group. MBL and properdin levels were comparable between the two groups at the start and at the end of the HD session. Conversely, at 30 min intradialysis, MBL levels decreased significantly in the event-free group but not in the CV-event group (*P* < 0.05). Furthermore, properdin levels were significantly lower at 30 min in the CV-event group, compared to the event-free group. To summarize, MBL consumption was seen in the event-free group implying LP activation, while lower properdin levels were observed in the CV-event group suggesting AP activation.

### Inflammatory and pro-thrombotic factors in the HD patients

**Figure 3:**
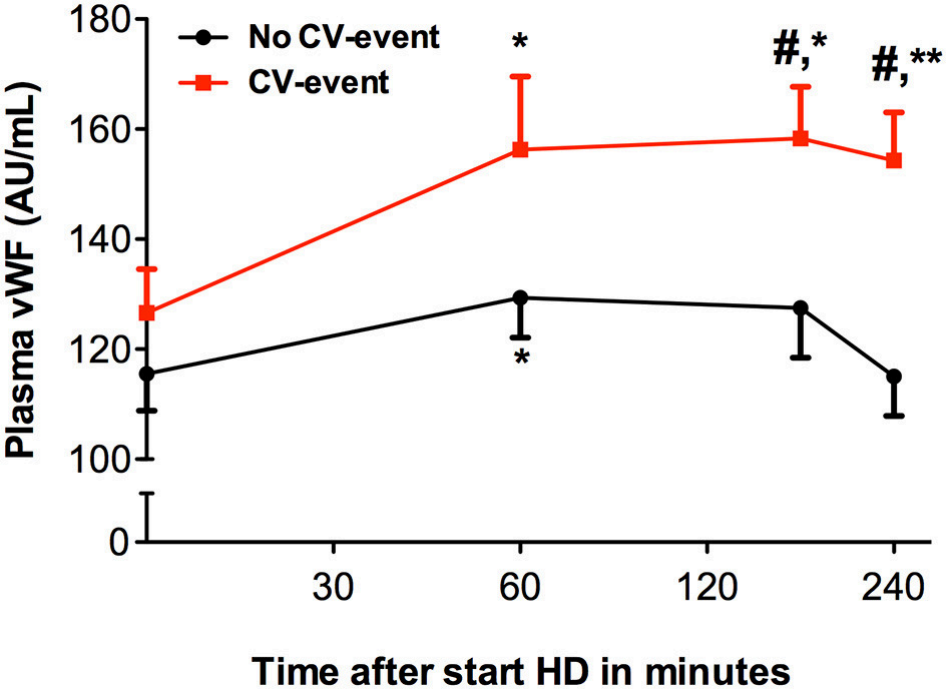
Levels of von Willebrand factor during hemodialysis. Course of von Willebrand factor (vWF) in patients that developed a cardiovascular event (CV-event) during follow-up and in those that remained CV-event free (no CV-event). The data is presented as mean ± SEM. vWF was determined at the start of hemodialysis session and 60, 180 and 240 min after the start of the session. Differences between the two groups were assessed by the student *t*-test and a one-way ANOVA followed by Bonferroni's *post-hoc* comparisons tests was used to compare levels at different time points within one group (*P < 0.05, **P < 0.01). The hashtag above the bars denotes a significant difference between the two groups (^#^*P* < 0.05), whereas the asterisk above the bars denotes a significant difference compared to baseline within the group. The number of subject is 17 in the “CV-event group” and 38 in the “No CV-event group”.

**Figure 4:**
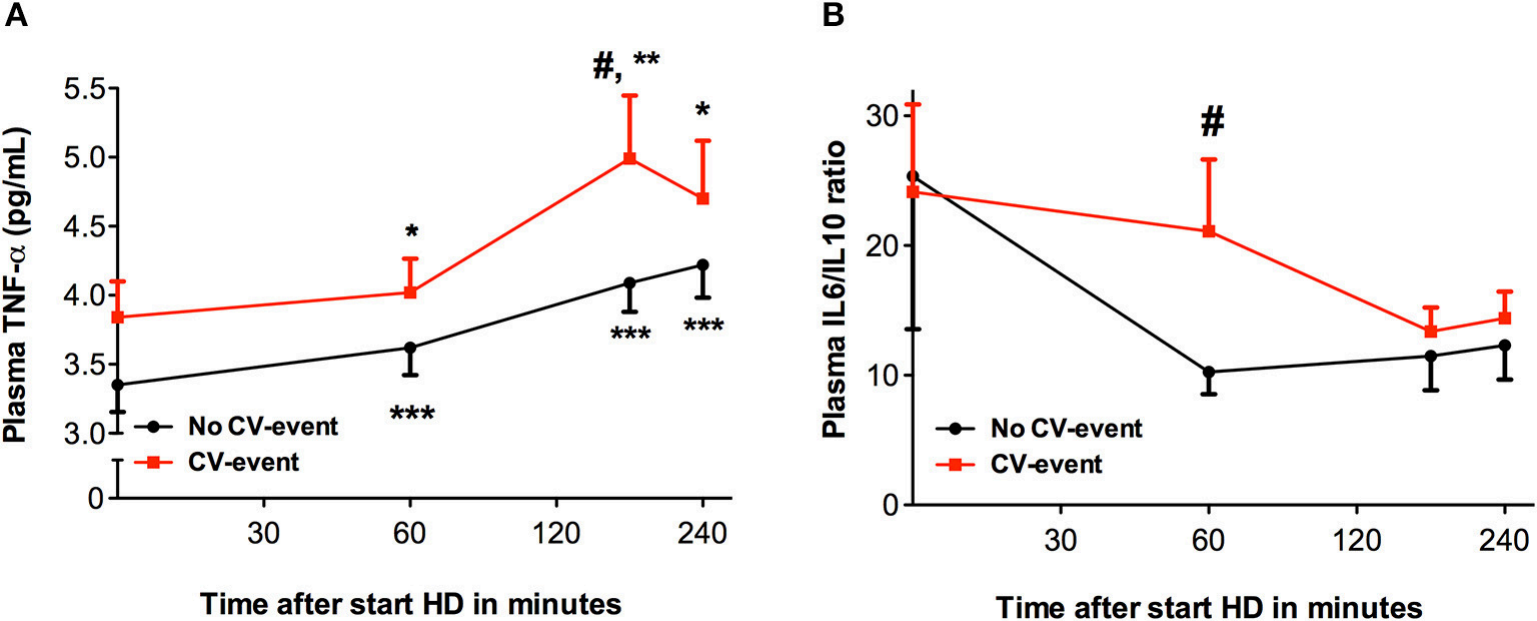
Levels of tumor necrose factor alpha and the ratio of interleukin-6 to interleukin-10 during hemodialysis. Course of tumor necrose factor alpha (TNF-α) and ratio of interleukin-6 (IL-6) to interleukin-10 (IL-10) in patients that developed a cardiovascular event (CV-event) during follow-up and in those that remained CV-event free (no CV-event). The data is presented as mean ± SEM. **(A)** The levels of TNF-α were determined at the start of hemodialysis session and 60, 180 and 240 min after the start of the session. **(B)** Levels IL-6 and IL-10 were determined at the start of hemodialysis session and 60, 180, and 240 min after. The IL-6/IL- 10 ratio was calculated by dividing the IL-6 (in pg/mL) values by the IL-10 levels (in pg/mL). Differences between the two groups were assessed by the student *t*-test and a one-way ANOVA followed by Bonferroni's *post-hoc* comparisons tests was used to compare levels at different time points within one group. (*P < 0.05, **P < 0.01, ***P < 0.001). The hashtag above the bars denotes a significant difference between the two groups (^#^*P* < 0.05), whereas the asterisk above the bars denotes a significant difference compared to baseline within the group. The number of subject is 17 in the “CV-event group” and 38 in the “No CV-event group”.

We determined cytokines and Von Willebrand factor (vWF) to investigate if complement activation during HD was accompanied by a pro-inflammatory response and a pro-thrombotic state. During HD, distinct time-courses for levels of vWF were observed between the two groups (Figure 3). In the CV-event group, vWF levels increased steadily during the session (*P* < 0.05). Furthermore, compared to the event-free group, the CV-event group had significantly higher levels of vWF at 180 and 240 min intradialysis (*P* < 0.05). Cytokines such as tumor necrosis factor-α (TNF-α) may initiate inflammation and are therefore believed to play a role in dialysis-related cardiovascular risk. Levels of TNF-α rose significantly during the HD session in both groups (Figure 4A). In the CV-event group, levels peaked at 180 min after the start of the HD session (*P* < 0.01) and were significantly higher than in the event-free group (*P* < 0.05). Furthermore, in the event-free group, the maximum TNF-α levels were reached at the end of the session (*P* < 0.001).

To evaluate the relation between anti-inflammatory cytokines and pro-inflammatory cytokines, we determined the IL-6/IL-10 ratio (Figure 4B). Interestingly, IL-6/IL-10 ratios were the highest in both groups at the start of the HD session and showed a decreasing trend during the dialysis session, although not significant compared to baseline. Moreover, at 60 min intradialysis an important decrease in the IL-6/IL-10 ratio occurred in the event-free group, indicating a shift toward a less inflammatory profile. However, IL-6/IL-10 ratios remained elevated in the HD patients that developed a CV-event during follow-up, revealing a significant difference between the groups at this time point (*P* < 0.05). Overall, enhanced levels of pro-inflammatory and pro-thrombotic mediators seem to prelude the development of CV-events in HD patients.

### *Ex-vivo* model of hemodialysis

**Figure 5:**
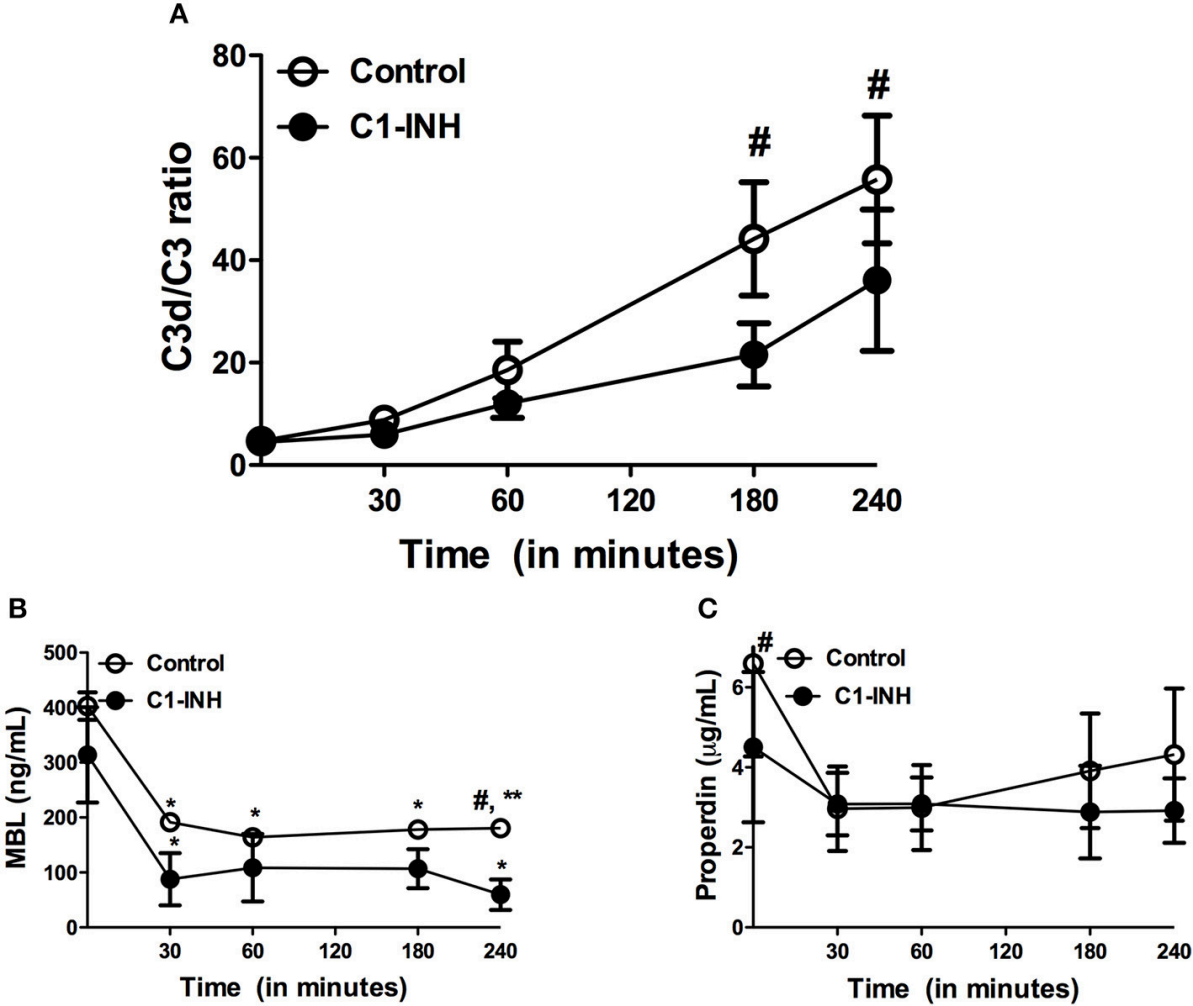
Complement levels during *ex vivo* hemodialysis. Two different sessions were performed with whole blood of three healthy donors; one session with C1-inhibitor (C1-INH) and one session without, the control session. The data is presented as mean ± SEM. **(A)** C3d/C3 ratios were measured to determine complement activation. **(B)** MBL levels significantly decrease over time during the session. **(C)** Properdin levels were reduced during the session, although not significantly. Differences between the two groups were assessed by the student *t*-test and a one-way ANOVA followed by Bonferroni's *post-hoc* comparisons tests was used to compare levels at different time points within one group (*P < 0.05, **P < 0.01). The hashtag above the bars denotes a significant difference between the two groups (^#^*P* < 0.05), whereas the asterisk above the bars denotes a significant difference compared to baseline within the group.

**Figure 6:**
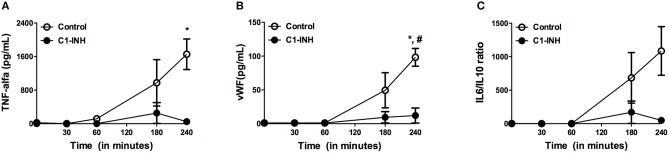
Levels of tumor necrose factor alpha, ratio of interleukin-6 to interleukin-10 and vWF during *ex vivo* hemodialysis. Two different sessions were performed with whole blood of three healthy donors; one session with C1-inhibitor (C1-INH) and one session without, the control session. The data is presented as mean ± SEM. **(A)** TNF-α levels significantly increased in the control session, but not in the session with C1-INH. **(B)** Correspondingly, vWF showed a significant rise in the control session, while C1 INH additionlead to significantly lower levels. **(C)** The IL-6/IL-10 ratio progressively increased over time, although not statistically significant. Differences between the two groups were assessed by the student *t*-test and a one-way ANOVA followed by Bonferroni's *post-hoc* comparisons tests was used to compare levels at different time points within one group (*P < 0.05). The hashtag above the bars denotes a significant difference between the two groups (^#^*P* < 0.05), whereas the asterisk above the bars denotes a significant difference compared to baseline within the group.

To further evaluate the effect of HD-induced complement activation on inflammation and coagulation, we used an *ex-vivo* model of HD. During the 4 h of perfusion, the C3d/C3 ratio increased progressively from 4.7 ± 0.6 at baseline to 55.8 ± 12.5 after 240 min (Figure 5A). MBL and properdin levels were determined to discriminate between complement activation via the AP and/or the LP. Both MBL and properdin levels decreased significantly over time. After 4 h, MBL levels were reduced by 55.2% (*P* < 0.05) and properdin levels by 34.4%, respectively (Figure 5). We next assessed inflammatory and pro-thrombotic factors. Similarly to complement activation, the HD model resulted in a significant increase in TNF-α, IL-6/IL-10 ratio, and vWF levels after 240 min of dialysis (Figure 6).

Finally, we evaluated the effect of complement inhibition in our model to test if complement activation acts as a trigger for inflammation and coagulation in HD. C1-INH was added and significantly reduced C3d/C3 ratios compared to controls, namely from 55.8 ± 13 to 33.1 ± 24 (Figure 5A; *P* < 0.05). HD-induced consumption of properdin and MBL was not prevented by the use of C1-INH.Furthermore, TNF-α levels were 1654 ± 631 ng/mL after 240 min in the control session, while in the session with C1-INH levels were reduced to 48.7 ± 74.7 ng/mL(Figure 6A). Correspondingly, a similar trend was seen after 240 min in the IL-6/IL-10 ratio (control session 1086 ± 630 pg/mL, C1-INH session 51 ± 78 pg/mL; Figure 6C) and for vWF levels (control session: 98.2 ± 22.7 pg/mL, C1-INH session: 1.7 ± 3.3 pg/mL) when the control session was compared to C1-INH session (Figure 6B). To summarize, C1-INH addition was able to inhibit HD-induced complement activation and thereby reduce vWF, TNF-α and IL-6/IL-10 by 98, 97, and 95% respectively.

## Discussion

Hemodialysis treatment comes with the balance between the dangers of advanced uremia and the inherent risks related to this form of RTT (22, 23). The higher cardiovascular risk seen in this population is not only related to ESRD but it is also associated with the HD procedure itself (2). Innate immunity has been proposed to be the missing link in the mechanism of CV-events in HD patients (4). We observed distinct differences in molecular profiles during HD of patients that will later develop CV-events compared to those who remained event-free during follow-up. At the start of dialysis, a unique peak in complement activation was only seen in patients in the CV-event group. Furthermore, enhanced inflammation and coagulation accompanied the complement activation seen in HD patient that will develop CV-events. Moreover, these processes arose long before the actual development of the CV-event. Altogether these three elements showed different dynamics, with complement activation possibly initiating these processes. In accordance, complement inhibition in our *ex-vivo* model not only decreased complement activation but also diminished pro-inflammatory and pro-thrombotic mediators.

Despite significant advances in the biocompatibility of HD membranes, complement activation remains an undesired but relevant issue (8, 15). Higher levels of complement components as well as loss of complement inhibitors have been associated with a higher risk for cardiovascular disease in HD patients (8, 11–14). Recently, complement activation prior to a HD session was associated with the occurrence of CV-events in HD patients (15). Here, we showed that activation of C3 during dialysis is linked to the development of CV-events. Our study is the first, to our knowledge, to assess the relationship between intradialytic complement activation and subsequent outcome. In accordance, previous studies have shown that activation of the complement system peaks during the first 15 to 30 min of the HD session (24). However, the mechanism by which complement activation increases the risk for cardiovascular disease remains largely unknown.

The LP and AP initiate complement activation during HD (25, 26). In our study, we only found MBL consumption in the event-free group, implying that this decrease is actually beneficial. In accordance, MBL has been proposed to be involved in the removal of atherogenic particles, thereby decreasing atherosclerosis. Our previous data showed that higher MBL levels in HD patients were associated with protection against cardiovascular disease (9). We also found a rise in properdin levels in the event-free group. Properdin, unlike other complement factors, is produced by leukocytes, predominately neutrophils (27). Therefore, the increase in properdin is presumably the result of leukocyte activation by the HD membrane leading to degranulation (28). Since, this rise was not seen in the CV-event group, we speculate this was due to properdin consumption by AP activation in these patients.

We found higher TNF-α levels and IL-6/IL-10 ratios in patients that would develop a CV-event. TNF-α and IL-6 are potent cytokines that can initiate a powerful pro-inflammatory reaction (29, 30). If this response is not contained, it can lead to hypotension, organ dysfunction, and eventually result in death. Elevated levels of these cytokines have also been related to an increased risk for CV-events in the general population and in HD patient (31–34). In contrast, IL-10 is a major anti-inflammatory cytokine with the ability to suppress the production and secretion of pro-inflammatory mediators in leukocytes, thereby effectively controlling the inflammation (35). The IL-6/IL-10 ratio has previously been linked to outcome after inflammatory disorders and to the development of HD-induced left ventricular dysfunction (36–38). In *ex-vivo* models, the induction of IL-6 during the bio-incompatibility reaction was shown to be completely complement-dependent, while the induction of TNF-α was only partially complement-dependent (16). In addition, in a primate model of HD, complement inhibition lead to enhanced levels of IL-10, demonstrating the relationship between the two systems (39).

Thrombosis is a key element in the development of cardiovascular disease. Previously, Péquériaux et al. reported that vWF is a good predictor of CV-events in patients undergoing RRT (40). Von Willebrand factor is a glycoprotein involved in hemostasis but vWF is also a marker of endothelial cell activation (41). We found significantly higher levels of vWF in the group of patients who developed CV-events, which could be evidence of a prothrombotic state. vWF is produced in endothelial cells and megakaryocytes, but also stored in the granules of platelets (42). Considering our *ex-vivo* lacks endothelial cells, the vWF is most likely derived from platelets. The release of vWF could either be the direct effect of complement activation or via C5a-activated leukocytes (43, 44). The link between the complement system and thrombosis is not new in HD (45). Complement receptors on leukocytes are important for the formation of platelet-leukocytes complexes, which contributes to thrombotic processes (46). In addition, complement activation during HD induces the production of pro-coagulation factors (47). Moreover, plasma levels of C3 correlated with a denser clot structure in HD patients (48).

The complement system is a strong mediator of the bioincompatibility reaction. Therefore, we proposed that complement activation has an essential role in orchestrating the inflammatory response in HD. In accordance with the result observed in the HD patients; our *ex-vivo* model demonstrated that the dialyzer induces complement activation, inflammation and enhances coagulation. We then wondered if HD-induced inflammation could be attenuated by complement inhibition. Addition of C1-INH to the *ex-vivo* HD model significantly diminished HD-induced complement activation and also almost completely abolished the induction of TNF-α levels, L-6/IL-10 ratios and vWF levels. Similarly, Kourtzelis et al, also demonstrated in an *ex vivo* model of HD the induction of coagulation during 2 h of perfusion. In their study, compstatin was used to block complement activation at the level of C3 (10). We postulate that HD-induced complement activation results in the formation of anaphylatoxins, thereby resulting in the activation of peripheral blood mononuclear cell and platelets initiating a pro-inflammatory and pro-thrombotic response. Previously, several reports demonstrated that bioincompatibility-induced inflammation relies mainly on complement, whereas granulocyte enzyme release was predominantly C3-dependent, leukocyte activation and pro-thrombotic mediators were largely dependent on C5 (16, 46). Altogether, our results support the hypothesis of the complement system as a key component in HD-induced inflammation and coagulation, which subsequently leads to a higher risk for CV-events.

We are aware that our study has strengths and limitations. Although the study has a long follow up, the samples were only collected during a single hemodialysis session. Our study could have benefited from a second assessment of these parameters during another dialysis session in the same patients, to assess reproducibility and increase reliability. Furthermore, while complement activation was seen in the patients during HD as well as in our *ex-vivo* model, clear differences were present. Moreover, in patients a significant peak of complement activation was seen during the first 30 min of HD. In contrast, in our *ex-vivo* model a continuous rise of complement activation was seen until the end of the session. Obviously these discrepancies arise due to the differences of *in*-*vivo* to *ex-vivo*. For instance, in the *ex-vivo* model blood recirculates without re-entering the human body, therefore it lacks the interaction with endothelial cells, liver and other organs. Lastly, the size of our cohort could be considered small and therefore might impact the statistical analysis. However, due to the long follow up, we achieved a relatively high number of CV-events which increases the power of the study in the comparisons between the CV-event group and the event free group.

There is a growing body of data supporting a role for the complement system in the development of cardiovascular disease. Ekdahl et al. proposed that complement activation initiates an inflammatory cascade and amplifies pro-thrombotic processes (4). For the first time, to our knowledge, we demonstrated intradialytic differences in complement activation, inflammation and a pro-thrombotic factor in HD patients that will develop a CV-event compared to HD patients that will not. Furthermore, we showed that complement inhibition during HD resulted in decreased levels of the pro-inflammatory and pro-thrombotic mediators. Future studies have to determine what the ideal target is to inhibit complement in HD to attenuate these processes and to determine if this decreases the risk of CV-events in HD patients.

## Author contributions

FP, MG, MD, CF, and MS research idea and study design. FP, MG and SA data acquisition. FP, MG, BF, SB, MD, JM, WvS, CF, and MS data analysis/interpretation. FP and MG statistical analysis and wrote the manuscript. All authors were involved in editing the final manuscript. All authors read and approved the final manuscript.

### Conflict of interest statement

The authors declare that the research was conducted in the absence of any commercial or financial relationships that could be construed as a potential conflict of interest.

## Abbreviations

AP: Alternative pathway
BSA: Body surface area
C5b-9: Membrane attack complex
CV-event: Cardiovascular event
ESRD: End stage renal disease
ELISA: Enzyme-linked immunosorbent assay
HD: Hemodialysis
IL-6: Interleukin 6
IL-10: Interleukin 10
LP: Lectin pathway
MBL: Mannose-binding lectin
RRT: Renal replacement therapy
PCI: Percutaneous coronary intervention
TNF-α: Tumor necrosis factor-α
vWF: Von Willebrand factor

## References

[1] Robinson BM Akizawa T Jager KJ Kerr PG Saran R Pisoni RL. Factors affecting outcomes in patients reaching end-stage kidney disease worldwide: differences in access to renal replacement therapy, modality use, and haemodialysis practices. Lancet (2016) 388:294–306. 10.1016/S0140-6736(16)30448-2 27226132 PMC6563337

[2] Assa S Hummel YM Voors AA Kuipers J Westerhuis R de Jong PE. Hemodialysis-induced regional left ventricular systolic dysfunction: prevalence, patient and dialysis treatment-related factors, and prognostic significance. Clin J Am Soc Nephrol. (2012) 7:1615–23. 10.2215/CJN.00850112 22822014 PMC3463203

[3] Weiner DE Tighiouart H Amin MG Stark PC MacLeod B Griffith JL. Chronic kidney disease as a risk factor for cardiovascular disease and all-cause mortality: a pooled analysis of community-based studies. J Am Soc Nephrol. (2004) 15:1307–15. 10.1097/01.ASN.0000123691.46138.E2 15100371

[4] Ekdahl KN Soveri I Hilborn J Fellström B Nilsson B. Cardiovascular disease in haemodialysis: role of the intravascular innate immune system. Nat Rev Nephrol. (2017) 13:285–96. 10.1038/nrneph.2017.17 28239169

[5] Nilsson B Ekdahl KN Mollnes TE Lambris JD. The role of complement in biomaterial-induced inflammation. Mol Immunol. (2007) 44:82–94. 10.1016/j.molimm.2006.06.020 16905192

[6] Ricklin D Hajishengallis G Yang K Lambris JD. Complement: a key system for immune surveillance and homeostasis. Nat Immunol. (2010) 11:785–97. 10.1038/ni.1923 20720586 PMC2924908

[7] Craddock PR Fehr J Brigham KL Kronenberg RS Jacob HS. Complement and leukocyte-mediated pulmonary dysfunction in hemodialysis. N Engl J Med. (1977) 296:769–774. 10.1056/NEJM197704072961401 840277

[8] Poppelaars F Gaya da Costa M Berger SP Assa S Meter-Arkema AH Daha MR Strong predictive value of mannose-binding lectin levels for cardiovascular risk of hemodialysis patients. J Transl Med. (2016) 14:236 10.1186/s12967-016-0995-5 27495980 PMC4974702

[9] Poppelaars F Gaya da Costa M Berger SP Assa S Meter-Arkema AH Daha MR. Erratum to: strong predictive value of mannose-binding lectin levels for cardiovascular risk of hemodialysis patients. J Transl Med. (2016) 14:236. 10.1186/s12967-016-1004-8 27495980 PMC4974702

[10] Kourtzelis I Markiewski MM Doumas M Rafail S Kambas K Mitroulis I. Complement anaphylatoxin C5a contributes to hemodialysis-associated thrombosis. Blood (2010) 116:631–9. 10.1182/blood-2010-01-264051 20424189 PMC3086498

[11] Buraczynska M Ksiazek P Wacinski P Zukowski P Dragan M Bednarek-Skublewska A. Complement receptor 1 gene polymorphism and cardiovascular disease in dialyzed end-stage renal disease patients. Hum Immunol. (2010) 71:878–82. 10.1016/j.humimm.2010.06.001 20538029

[12] Buraczynska M Ksiazek P Zukowski P Benedyk-Lorens E Orlowska-Kowalik G. Complement factor H gene polymorphism and risk of cardiovascular disease in end-stage renal disease patients. Clin Immunol. (2009) 132:285–90. 10.1016/j.clim.2009.04.005 19428307

[13] Kishida K Kishida N Arima M Nakatsuji H Kobayashi H Funahashi T. Serum C1q- binding adiponectin in maintenance hemodialysis patients. BMC Nephrol. (2013) 14:50. 10.1186/1471-2369-14-50 23442371 PMC3598349

[14] Satomura A Endo M Fujita T Ohi H Ohsawa I Fuke Y. Serum mannose-binding lectin levels in maintenance hemodialysis patients: impact on all-cause mortality. Nephron Clin Pract. (2006) 102:c93–9. 10.1159/000089666 16282701

[15] Lines SW Richardson VR Thomas B Dunn EJ Wright MJ Carter AM. Complement and cardiovascular disease - the missing link in haemodialysis patients. Nephron (2015) 132:5–14. 10.1159/000442426 26695077

[16] Lappegard KT Christiansen D Pharo A Thorgersen EB Hellerud BC Lindstad J. Human genetic deficiencies reveal the roles of complement in the inflammatory network: lessons from nature. Proc Natl Acad Sci USA. (2009) 106:15861–6. 10.1073/pnas.0903613106 19717455 PMC2732707

[17] Assa S Gansevoort RT Westerhuis R Kobold ACM Voors AA de Jong PE. Determinants and prognostic significance of an intra-dialysis rise of cardiac troponin I measured by sensitive assay in hemodialysis patients. Clin Res Cardiol. (2013) 102:439–45. 10.1007/s00392-013-0551-8 23397594

[18] Rodríguez-Sanz A Sánchez-Villanueva R Domínguez-Ortega J Fiandor AM Ruiz MP Trocoli F. Mechanisms involved in hypersensitivity reactions to polysulfone hemodialysis membranes. Artif Organs. (2017) 41:E285–95. 10.1111/aor.12954 28722144

[19] Hempel JCJC Poppelaars F Gaya Da Costa M Franssen CFMCFM De Vlaam TPGTPG Daha MRMR. Distinct *in vitro* complement activation by various intravenous iron preparations. Am J Nephrol. (2017) 45:49–59. 10.1159/000451060 27889746

[20] Damman J Seelen MA Moers C Daha MR Rahmel A Leuvenink HG. Systemic complement activation in deceased donors is associated with acute rejection after renal transplantation in the recipient. Transplantation (2011) 92:163–9. 10.1097/TP.0b013e318222c9a0 21677599

[21] Austin PC Steyerberg EW. Interpreting the concordance statistic of a logistic regression model: relation to the variance and odds ratio of a continuous explanatory variable. BMC Med Res Methodol. (2012) 12:82. 10.1186/1471-2288-12-82 22716998 PMC3528632

[22] Slinin Y Greer N Ishani A MacDonald R Olson C Rutks I. Timing of dialysis initiation, duration and frequency of hemodialysis sessions, and membrane flux: a systematic review for a KDOQI clinical practice guideline. Am J Kidney Dis. (2015) 66:823–36. 10.1053/j.ajkd.2014.11.031 26498415

[23] Mcintyre CW Rosansky SJ Starting dialysis is dangerous: how do we balance the risk? Kidney Int. (2012) 82133:382–7. 10.1038/ki.2012.133 22534960

[24] Chenoweth DE. Complement activation during hemodialysis: clinical observations, proposed mechanisms, and theoretical implications. Artif Organs. (1984) 8:281–90. 10.1111/j.1525-1594.1984.tb04291.x 6332607

[25] DeAngelis RA Reis ES Ricklin D Lambris JD. Targeted complement inhibition as a promising strategy for preventing inflammatory complications in hemodialysis. Immunobiology (2012) 217:1097–105. 10.1016/j.imbio.2012.07.012 22964235 PMC3439808

[26] Mares J Richtrova P Hricinova A Tuma Z Moravec J Lysak D. Proteomic profiling of blood-dialyzer interactome reveals involvement of lectin complement pathway in hemodialysis-induced inflammatory response. Proteomics Clin Appl. (2010) 4:829–38. 10.1002/prca.201000031 21137026

[27] Lubbers R van Essen MF van Kooten C Trouw LA. Production of complement components by cells of the immune system. Clin Exp Immunol. (2017) 188:183–94. 10.1111/cei.12952 28249350 PMC5383442

[28] Schmaldienst S Hö WH Degranulation of polymorphonuclear leukocytes by dialysis membranes—the mystery clears up? mediators involved in neutrophil degranulation include. Nephrol Dial Transpl. (2000) 15:1909–10. 10.1093/ndt/15.12.1909 11096129

[29] Kalliolias GD Ivashkiv LB. TNF biology, pathogenic mechanisms and emerging therapeutic strategies. Nat Rev Rheumatol. (2015) 12:49–62. 10.1038/nrrheum.2015.169 26656660 PMC4809675

[30] Hunter CA Jones SA. IL-6 as a keystone cytokine in health and disease. Nat Immunol. (2015) 16:448–57. 10.1038/ni.3153 25898198

[31] Pai JK Pischon T Ma J Manson JE Hankinson SE Joshipura K. Inflammatory markers and the risk of coronary heart disease in men and women. N Engl J Med. (2004) 351:2599–610. 10.1056/NEJMoa040967 15602020

[32] Zimmermann J Herrlinger S Pruy A Metzger T Wanner C. Inflammation enhances cardiovascular risk and mortality in hemodialysis patients. Kidney Int. (1999) 55:648–58. 10.1046/j.1523-1755.1999.00273.x 9987089

[33] Barreto DV Barreto FC Liabeuf S Temmar M Lemke HD Tribouilloy C. Plasma interleukin-6 is independently associated with mortality in both hemodialysis and pre-dialysis patients with chronic kidney disease. Kidney Int. (2010) 77:550–6. 10.1038/ki.2009.503 20016471

[34] Stenvinkel P Ketteler M Johnson RJ Lindholm B Pecoits-Filho R Riella M. IL-10, IL-6, and TNF-α: central factors in the altered cytokine network of uremia—The good, the bad, and the ugly. Kidney Int. (2005) 67:1216–33. 10.1111/j.1523-1755.2005.00200.x 15780075

[35] Ouyang W Rutz S Crellin NK Valdez PA Hymowitz SG. Regulation and functions of the IL-10 family of cytokines in inflammation and disease. Annu Rev Immunol. (2011) 29:71–109. 10.1146/annurev-immunol-031210-101312 21166540

[36] Assa S Hummel YM Voors AA Kuipers J Westerhuis R Groen H. Hemodialysis-induced regional left ventricular systolic dysfunction and inflammation: a cross-sectional study. Am J Kidney Dis. (2014) 64:265–73. 10.1053/j.ajkd.2013.11.010 24364893

[37] Sapan HB Paturusi I Jusuf I Patellongi I Massi MN Pusponegoro AD. Pattern of cytokine (IL-6 and IL-10) level as inflammation and anti-inflammation mediator of multiple organ dysfunction syndrome (MODS) in polytrauma. Int J Burns Trauma. (2016) 6:37–43. 27335696 PMC4913232

[38] Ng PC Li K Wong RPO Chui K Wong E Li G. Proinflammatory and anti-inflammatory cytokine responses in preterm infants with systemic infections. Arch Dis Child Fetal Neonatal Ed. (2003) 88:209–13. 10.1136/fn.88.3.F209 12719394 PMC1721542

[39] Reis ES DeAngelis RA Chen H Resuello RRG Ricklin D Lambris JD. Therapeutic C3 inhibitor Cp40 abrogates complement activation induced by modern hemodialysis filters. Immunobiology (2015) 220:476–82. 10.1016/j.imbio.2014.10.026 25468722 PMC4355228

[40] Pequeriaux NC Fijnheer R Gemen EF Barendrecht AD Dekker FW Krediet RT. Plasma concentration of von Willebrand factor predicts mortality in patients on chronic renal replacement therapy. Nephrol Dial Transplant. (2012) 27:2452–7. 10.1093/ndt/gfr735 22189209

[41] Sioulis A Malindretos P Makedou A Makris P Grekas D. Coagulation factors as biological risk markers of endothelial dysfunction. Association with the thrombotic episodes of chronic hemodialysis patients. Hippokratia (2009) 13:237–41. 20011089 PMC2776338

[42] Lenting PJ Christophe OD Denis CV. von Willebrand factor biosynthesis, secretion, and clearance: connecting the far ends. Blood (2015) 125:2019–28. 10.1182/blood-2014-06-528406 25712991

[43] Gralnick HR Williams SB McKeown LP Magruder L Hansmann K Vail M. Platelet von Willebrand factor. Mayo Clin Proc. (1991) 66:634–40. 10.1016/S0025-6196(12)60524-2 2046403

[44] Stokes KY Granger DN. Platelets: a critical link between inflammation and microvascular dysfunction. J Physiol. (2012) 590:1023–34. 10.1113/jphysiol.2011.225417 22183721 PMC3381810

[45] Amara U Rittirsch D Flierl M Bruckner U Klos A Gebhard F Lambris JD Huber-Lang M. Interaction between the coagulation and complement system. Adv Exp Med Biol. (2008) 632:71–9. 10.1007/978-0-387-78952-1_6 19025115 PMC2713875

[46] Bergseth G Lambris JD Mollnes TE Lappegård KT. Artificial surface-induced inflammation relies on complement factor 5: proof from a deficient person. Ann Thorac Surg. (2011) 91:527–33. 10.1016/j.athoracsur.2010.10.084 21256307 PMC3123536

[47] Innes A Farrell AM Burden RP Morgan AG Powell RJ. Complement activation by cellulosic dialysis membranes. J Clin Pathol. (1994) 47:155–8. 10.1136/jcp.47.2.155 8132830 PMC501832

[48] Schuett K Savvaidis A Maxeiner S Lysaja K Jankowski V Schirmer SH. A potent mortality risk factor in patients on hemodialysis. J Am Soc Nephrol. (2017) 28:1622–30. 10.1681/ASN.2016030336 28057772 PMC5407718

